# Evaluating Artificial Intelligence Models for ICU Length of Stay Prediction: A Systematic Review and Meta-Analysis

**DOI:** 10.3390/healthcare14091131

**Published:** 2026-04-23

**Authors:** Carlos Zepeda-Lugo, Andrea Insfran-Rivarola, Marcos Sanchez-Lizarraga, Sharon Macias-Velasquez, Ana-Pamela Arevalos, Yolanda Baez-Lopez, Diego Tlapa

**Affiliations:** 1 Facultad de Ingeniería, Arquitectura y Diseño, Universidad Autónoma de Baja California, Ensenada 21100, Mexico; czepeda@uabc.edu.mx; 2 Laboratorio de Producción y Métodos, Departamento de Ingeniería Industrial, Universidad Nacional de Asunción, San Lorenzo 2160, Paraguay; andrea.insfran@ing.una.py (A.I.-R.); aarevalos@fiuna.edu.py (A.-P.A.); 3 Unidad Académica de Negocios, Universidad Autónoma de Sinaloa, Los Mochis 81223, Mexico; marcos.sanchez@uas.edu.mx; 4 Facultad de Ingeniería, Universidad Autónoma de San Luis Potosí, San Luis Potosí 78290, Mexico; sharon.macias@uaslp.mx

**Keywords:** intensive care, length of stay, machine learning, deep learning, health informatics, sustainability

## Abstract

**Highlights:**

**What are the main findings?**

- ML and DL models demonstrated high predictive performance for ICU LOS.
- Predictive accuracy is consistent across ICU types, indicating robust generalizability of admission-time clinical data.

**What are the implications of the main findings?**

- AI-based LOS prediction can support early clinical decision-making, improving patient stratification and care planning.
- Reliable forecasts of ICU demand can enhance more efficient and sustainable healthcare systems.

**Abstract:**

**Background/Objectives:** Efficient management of intensive care unit (ICU) resources is a critical challenge for modern healthcare systems, which must balance high-quality patient care with operational and financial performance. ICU length of stay (LOS) is a key metric of clinical complexity and hospital efficiency. However, traditional methods for predicting LOS often fail to capture the complex, nonlinear interactions among physiological, demographic, and treatment-related variables. Machine learning (ML) and deep learning (DL) models have emerged as promising tools for enhancing predictive accuracy and supporting data-driven decision-making. **Methods:** This study presents a systematic review and meta-analysis of ML and DL approaches for predicting ICU LOS in adult patients. Following PRISMA guidelines, eight scientific databases were searched, yielding 33 eligible studies published between 2015 and 2025. **Results:** Mixed medical–surgical ICUs were the most common setting (51.5%), and 45.5% of datasets were sourced from public repositories. Most studies (19/33) focused on binary classification of prolonged stays, although thresholds ranged from >48 h to ≥14 days. The pooled results from ten studies yielded an AUROC of 0.9005 (95% CI: 0.8890–0.9121), indicating strong predictive capability across diverse clinical contexts. Subgroup analyses showed comparable performance between specialized surgical and general ICUs. **Conclusions:** These findings suggest that AI-driven LOS prediction models exhibit strong discriminatory power for ICU LOS prediction, supporting hospital capacity planning. However, to translate this into reliable clinical support, the methodological heterogeneity, scarcity of external validation, and near absence of calibration reporting identified in this review need to be addressed.

## 1. Introduction

Healthcare systems worldwide face increasing pressure to deliver high-quality care while maintaining efficiency. Intensive care units (ICUs) represent one of the most resource-intensive components of hospital systems, accounting from 13.4% [1] up to 20% [2] of total hospital costs. Length of stay (LOS) in the ICU refers to the duration of time from when a patient is admitted to discharge [3,4]. This metric is crucial for clinical as well as operational healthcare management [5,6]. Clinically, ICU LOS reflects the severity of illness, complexity of treatment, and recovery trajectory of critically ill patients suffering from conditions such as severe infections, trauma, or postoperative complications [7,8]. Thus, ICU LOS is often related to mortality risk and long-term outcomes [4,9]. Operationally, ICU LOS is directly related to capacity planning, resource allocation, and cost estimation [10,11]. Prolonged ICU stays may lead to bed shortages and delayed admission, affecting both patients and hospital systems. Therefore, accurate prediction of ICU LOS is essential for improving clinical decision-making and supporting efficient healthcare delivery through optimized resource allocation.

Traditionally, ICU LOS prediction has relied on rule-based systems or classical statistical models such as linear regression and survival analysis [4,12]. While these approaches have provided useful insights by capturing nonlinear relationships at a basic level [13], they often struggle to capture complex nonlinear relationships among physiological variables and clinical factors [14], reducing their practical value for decision support in modern hospitals.

Recent advances in digital healthcare technologies have enabled the utilization of machine learning (ML) methods capable of extracting patterns from large clinical datasets and predicting ICU LOS [15,16]. Algorithms such as decision trees (DT), random forests (RF), support vector machines (SVM), and gradient boosting methods (GB) have shown improved performance in dealing with the multi-faceted nature of ICU data [3], mainly due to their ability to model non-linear relationships between variables, complex interactions between predictors, and improve generalization to diverse patient datasets for uncompromised LOS prediction capabilities [3,17]. Publicly available large-scale clinical databases, including the Medical Information Mart for Intensive Care (MIMIC), and the electronic ICU (eICU) Collaborative Research [4,18] have further accelerated the development and evaluation of predictive models.

Similarly, over the past few years, advanced deep learning (DL) methods have been used to predict ICU LOS [19]. Architectures such as long short-term memory (LSTM) networks, attention-based models, and transformers have demonstrated potential in modeling the temporal dynamics of patient trajectories [20,21,22]. These models excel at sequential data processing, making them ideal candidates for examining time-series vital signs, laboratory values, and intervention records [23,24,25]. DL methods enable the combination of any feature, like static demographic and dynamic clinical features, to predict ICU LOS more accurately [4,18].

The commonly used performance metrics to evaluate LOS prediction models include the area under the receiver operating characteristic curve (AUROC), which is among the most cited in the literature [4,18,26]. AUROC measures how well a model distinguishes between short and long LOS across various classification thresholds [27,28]. This metric is especially useful for ICU LOS prediction, where class imbalance is common, as it measures performance without regard to any particular threshold [28,29]. In this context, AUROC values can have a range of 0.5 (guessing randomly) to 1.0 (perfect discrimination) [30,31]. Complementary metrics such as sensitivity, specificity, and calibration provide useful insights into the performance of the model in clinical practice [3,4,18,21].

From a healthcare management perspective, predictive analytics in healthcare has the potential to contribute to more resilient and efficient health systems. By anticipating ICU demand and patient trajectories, hospitals can reduce unnecessary resource consumption, improve bed turnover rates, and minimize operational inefficiencies. Recent evidence indicates that such predictive tools can support bed management, staffing optimization, and early discharge planning, thereby improving patient throughput and reducing avoidable delays in elective admissions [32]. However, translating predictive accuracy into measurable improvements in care delivery requires prospective validation, integration into clinical workflows, and systematic evaluation of impact on patient-relevant outcomes [33].

Despite the growing number of studies proposing ML and DL models for ICU LOS prediction, several important gaps remain in the literature [3,34,35]. Existing studies vary widely in terms of model architecture, dataset characteristics, outcome definitions, and evaluation metrics, making it difficult to assess the overall reliability and generalizability of these approaches. Additionally, many studies report performance metrics using heterogeneous methodologies, limiting direct comparisons across models and clinical contexts.

To address these challenges, this study conducts a systematic review and meta-analysis of published research evaluating ML and DL models for predicting ICU LOS in adult patients. By synthesizing evidence across multiple datasets and methodological approaches, this work aims to provide a comprehensive overview of the current state of artificial intelligence applications for ICU LOS prediction and their potential contribution to sustainable critical care management. This study seeks to address the following research questions.

RQ1. What is the overall predictive performance of ML and DL models for ICU LOS prediction across different clinical datasets and healthcare settings?

RQ2. How do methodological characteristics—including model architecture, validation strategy, and feature engineering—affect the predictive performance and reliability of ICU LOS prediction models?

RQ3. What methodological trends, reporting practices, and sources of bias can be identified in the development and evaluation of ICU LOS prediction models?

RQ4. How can AI-based ICU LOS prediction models contribute to improving operational efficiency and supporting sustainable resource management in critical care systems?

Unlike prior reviews that primarily focus on mortality prediction or provide narrative summaries of machine learning applications in critical care, this study specifically targets ICU length of stay (LOS) prediction using admission-time data and includes a quantitative meta-analysis of model discrimination performance (AUROC), enabling a more robust comparison across heterogeneous studies. In addition, this review explicitly frames ICU LOS prediction within the context of sustainable healthcare, highlighting its relevance for resource optimization, capacity planning, and operational efficiency.

The remainder of this paper is organized as follows: Section 2 describes the methodology adopted for this systematic review and meta-analysis. The summary of results is presented in Section 3. Section 4 widely discusses our results. Finally, limitations and conclusions are presented in Section 4 and Section 5, respectively.

## 2. Materials and Methods

### 2.1. Study Design and Protocol Registration

This study was conducted as a systematic review and meta-analysis following the Preferred Reporting Items for Systematic Reviews and Meta-Analyses (PRISMA) guidelines [36]. The review protocol was prospectively registered in the International Prospective Register of Systematic Reviews (PROSPERO; Ref. CRD420251089785). The complete PRISMA checklist is available in Supplementary Table S1.

### 2.2. Eligibility Criteria

Studies were selected according to predefined inclusion and exclusion criteria based on the Population–Predictor–Outcome–Study design (PPOS) framework.

Population. Studies involving adult patients (≥18 years) admitted to intensive care units were included. Eligible ICU settings comprised general, medical, surgical, trauma, cardiothoracic, and mixed medical–surgical ICUs [37].

Predictors. Studies using machine learning (ML) or deep learning (DL) algorithms to predict length of stay based on variables available at or near the time of ICU admission were included [38].

Outcome. The primary outcome was ICU length of stay (LOS). Studies reporting LOS as a continuous variable, a categorical variable, or a binary classification of prolonged stay were considered eligible. Studies focusing exclusively on hospital LOS outside the ICU, pediatric populations, neonatal care, or obstetric patients were excluded.

Study Design. Eligible study designs included: retrospective cohort studies, prospective cohort studies, registry-based studies, and analyses using publicly available datasets (e.g., MIMIC, eICU).

Exclusion. We excluded review articles, editorials, conference abstracts without full manuscripts, book chapters, and non-English publications. We also excluded reports focusing on pediatrics, neonatal care, or obstetrics, as well as those that did not address an ICU LOS outcome. The inclusion criteria are detailed in Supplementary Table S2.

### 2.3. Information Sources and Search Strategy

We systematically searched eight electronic databases: PubMed, Web of Science, EBSCOhost (CINAHL), Elsevier, Emerald, IEEE Xplore, SCOPUS, and Springer. The initial search was conducted in September 2024, and the final update was completed in April 2025. Reference lists of included publications were manually examined to identify additional eligible studies. A gray literature search was also performed using key terms in Google Scholar to reduce publication bias. Search queries combined controlled vocabulary (e.g., MeSH terms such as “Intensive Care Units” and “Length of Stay”) with free-text keywords (e.g., “machine learning”, “deep learning”, “prediction”, “ICU”) to maximize retrieval sensitivity. We limited inclusion to English-language studies published between 2015 and 2025 to reflect the emergence and consolidation of ML/DL models for ICU-related prediction. Complete search strings are detailed in Supplementary Table S3.

### 2.4. Study Selection and Data Extraction

Study selection was carried out in two phases by two independent reviewers using a standardized Excel tracking sheet. The first phase involved titles and abstracts screening to exclude clearly irrelevant studies. The second phase involved a full-text assessment of potentially eligible articles according to predefined inclusion and exclusion criteria. Discrepancies at any stage were resolved by consensus, and reasons for exclusion at the full-text level were documented.

Data extraction was conducted independently and in duplicate using a customized spreadsheet based on the CHARMS and PROBAST frameworks [39]. Extracted information included study identifiers (first author, year), country, setting, study design, data source, study period, sample size, ICU LOS definition, algorithm family, feature selection method, approach to missing data, validation strategy, primary performance metric, additional classification metrics, continuous prediction metrics, and calibration measures. When multiple algorithms were reported in a study, the best-performing model, as measured by AUROC, was selected for synthesis. All disagreements in data extraction were resolved through discussion.

### 2.5. Risk of Bias Assessment

Risk of bias was assessed using the PROBAST+AI tool [40], which is specifically designed to evaluate prediction models using artificial intelligence. Each study was evaluated across four domains: participants (D1), predictors (D2), outcome (D3), and analysis (D4). Each domain was classified as low, high, or unclear risk of bias. Judgments and supporting rationale were documented in a standardized format. An overall risk of bias judgment was also assigned in accordance with PROBAST+AI guidance. Assessments were performed independently by two reviewers, with disagreements resolved by consensus. Results are summarized in Supplementary Table S4.

### 2.6. Data Synthesis and Meta-Analysis

For the systematic review, we created a descriptive summary of the included studies and a performance summary of the DL and ML models with the best metrics in each study. In addition, a meta-analysis was conducted to synthesize the discrimination performance of ML/DL models predicting ICU LOS, using the AUROC as the primary summary metric. Only studies that reported AUROC values with sufficient information to estimate their variance were included in the quantitative synthesis. This approach ensures that each study contributes a single, statistically independent effect size to the pooled analysis, preserving the assumptions of the random-effects model. Including multiple correlated models from the same dataset would violate the independence assumption and artificially inflate the precision of pooled estimates. Furthermore, selecting the best-performing model reflects the maximum demonstrated clinical potential of AI in each study context, aligning with the clinical focus of this review.

Given the expected heterogeneity in patient populations, ICU types, data sources, and modeling approaches, a random-effects model was selected to account for both within-study and between-study variability. This approach assumes that true effect sizes may differ across studies due to clinical and methodological diversity. Additionally, subgroup analyses were conducted based on ICU type (specialized surgical vs. general ICUs) to explore potential sources of heterogeneity.

Pooled AUROCs and their 95% confidence intervals (CI) were estimated using a generic inverse variance approach, with between-study variance computed via the restricted maximum likelihood (REML) method. To improve the accuracy and robustness of CI around pooled estimates, Hartung–Knapp method adjustments were applied [41,42,43]. Heterogeneity was assessed using the chi-square and *I^2^* statistic. Qualitatively, *I^2^* values of <25%, 25% to 75%, and >75% were considered low, moderate, and high levels of between-study variability in effect estimates, respectively [42]. A forest plot was created to display individual and pooled estimates. All computations ran in R (metafor 4.2-0), and Egger’s regression was used to examine funnel-plot asymmetry [44]. Additionally, a subgroup meta-analysis was conducted to compare specialized surgical ICUs with general ICUs. Finally, the full dataset of the included studies in the meta-analysis is depicted in Supplementary Table S5.

## 3. Results

### 3.1. Study Selection

**Figure 1:**
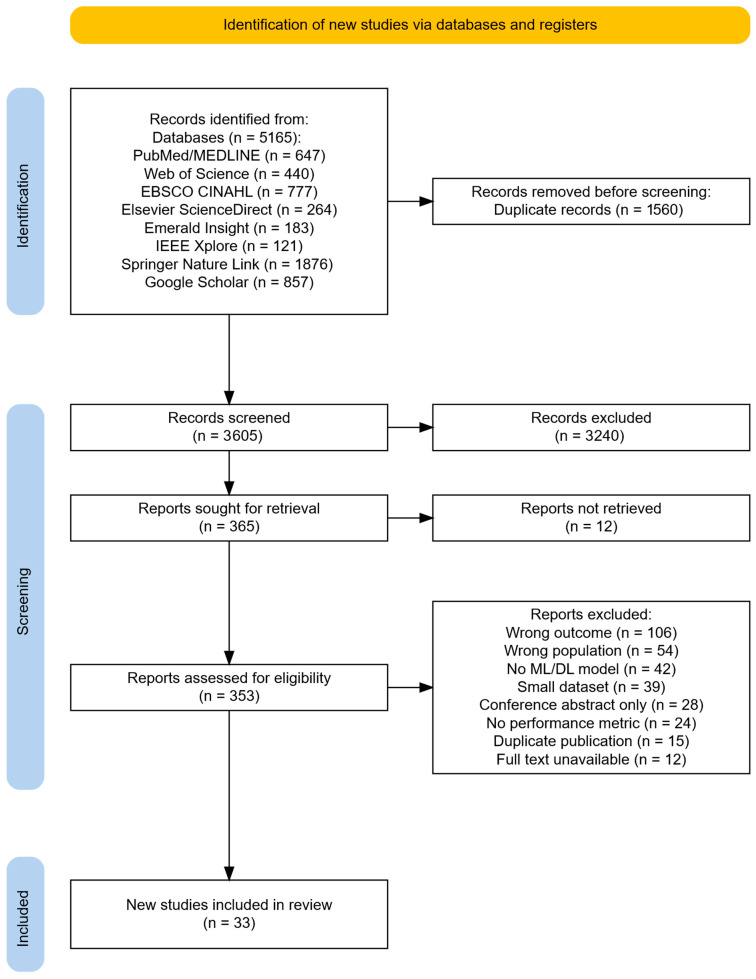
PRISMA study selection flow diagram.

The systematic search retrieved 5165 records from electronic databases. After removing 1560 duplicates, 3605 titles and abstracts were screened for eligibility criteria, resulting in 3240 exclusions. Full texts were sought for the remaining 365 records; however, 332 did not meet some of the criteria depicted in the PRISMA flow diagram [45] of Figure 1, resulting in 33 studies included in the qualitative synthesis, of which 10 contributed to the quantitative meta-analysis. Although the initial search identified a large body of literature, the final inclusion of 33 studies reflects the application of stringent eligibility criteria aimed at ensuring methodological consistency and comparability. The last search of the databases and registers was conducted in April 2025.

### 3.2. Study Characteristics

Table 1 presents the main characteristics of the studies included in the systematic review. The 33 included studies were conducted across 15 countries, with the United States (24.2%) and China (21.2%) contributing the most publications. The remaining studies were distributed across several regions, reflecting the global interest in applying ML and DL models to critical care prediction tasks.

Most studies were conducted in mixed adult ICUs, comprising 51.5% (17/33) of the sample. ICU settings accounted for 28/33 (84.8%) of the studies, with 5/33 (15.2%) conducted in related hospital environments.

Regarding data sources, publicly available datasets—particularly the MIMIC database—were widely used, contributing 45.5% of the analyzed datasets. The remaining studies relied primarily on institutional electronic health records (EHR) from single clinical centers. Sample size varied substantially across studies, ranging from 48 to 216,280 patients, reflecting differences in study design and data availability. Most studies employed retrospective observational designs using EHR data.

### 3.3. Outcome Definitions and Prediction Tasks

The operationalization of ICU LOS varied considerably across studies. Among the included investigations, 25 studies (75.8%) explicitly predicted ICU LOS outcomes. A total of 19 studies (57.6%) framed the prediction problem as a binary classification task, typically distinguishing between prolonged and non-prolonged stays. The commonly used thresholds to define a prolonged ICU stay were ≥7 days (26.3% of studies) and >5 days (10.5% of studies). Other studies treated ICU LOS as a continuous variable or as multi-category ordinal outcomes, resulting in heterogeneity in outcome definitions across the literature.

### 3.4. Performance of Machine and Deep Learning Models for ICU LOS

Among the leading algorithms in each study, random forests were the most common, achieving the highest performance in 14/33 studies (42%), followed by logistic regression in 6/33 studies (18%), and deep neural networks in 6/33 studies (18%).

Validation predominantly relied on hold-out splits (26/33), typically as a single split, sometimes combined with k-fold cross-validation, and only rarely paired with an external cohort; pure resampling without an independent split was infrequent. The most common validation strategy was a 70–80/30–20 hold-out split, applied in 16/33 (48.5%) studies. External validation was rarely conducted, with only 3 out of 33 studies (9.1%) evaluating model performance on independent datasets from different institutions or populations.

Feature handling varied. Some studies retained all predictors, others used LASSO, expert-specified or engineered sets, or end-to-end/autoML pipelines; additional approaches included missingness-threshold filters, correlation/RFE/stepwise procedures, and occasional information-gain ranking. Missing-data strategies were heterogeneous (and often under-reported), ranging from simple single-imputation (mean/median/mode) and complete-case analyses to MICE, threshold-based deletion, forward-fill with sentinel values, or bespoke methods. Discrimination (AUROC) was reported in 16/33 studies, spanning approximately 0.72 to 0.98; supplementary metrics appeared variably (e.g., accuracy, F1 score, sensitivity/specificity, R^2^, MAE, RMSE, PR-AUC), while calibration was seldom documented. Detailed per-study models, validation schemes, feature pipelines, missing-data handling, and exact estimates are provided in Table 2.

Most studies (75%) were published between 2021 and 2025. This period displayed the broadest methodological diversity. In contrast, the early phase (2015–2020) accounted for 25% of studies, with less methodological diversity. Across all studies, Tree-based ML is the most frequently reported family, appearing in 11 investigations (33.3%). Traditional regression and Ensemble boosting each account for 6 studies (18.2%), jointly representing 12 of 33 investigations (36.4%).

**Figure 2:**
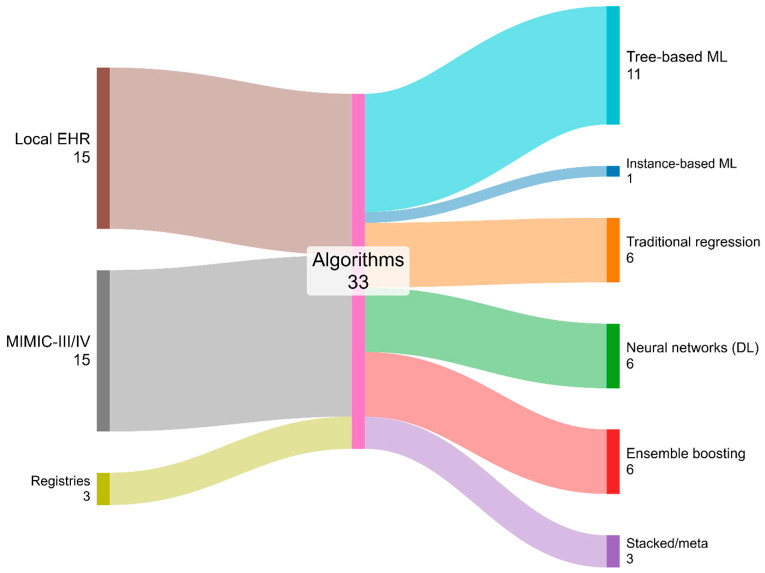
Data source to model workflows in ICU LOS Prediction Studies.

Deep learning neural network architectures (including fully connected networks, convolutional networks, autoencoder-based models, and transformer-based models) are reported in 6 studies (18.2%). Stacked/meta-approaches appear in 3 studies (9.1%), and Instance-based ML occurs once (3.0%). Aggregating across families, Tree-based ML plus Ensemble boosting sum to 17 studies (51.5%), indicating that decision tree-derived paradigms are the most widely used family. In contrast, deep learning neural network architectures and Stacked/meta-approaches together account for 9 studies (27.3%), reflecting a smaller yet nontrivial proportion of investigations adopting advanced representation learning or combination strategies. Local institutional EHR datasets contributed 15 studies (45.5%), MIMIC-III or IV contributed 15 (45.5%), and external registries contributed 3 (9.1%). Figure 2 supports inference about marginal distributions by data source and by modeling family for the 33 included studies, while the intermediate “Algorithms” node indicates that source-by-family cross-tabulations are not encoded in this visualization and would require a dedicated cross-table to be reported.

### 3.5. Risk of Bias

Risk of bias was assessed using the PROBAST+AI framework, which evaluates prediction models across four domains: participants, predictors, outcome, and analysis.

Across the included studies, methodological limitations were primarily identified in the analysis domain, reflecting issues with model validation strategies, insufficient reporting of calibration metrics, and limited use of external validation datasets. Only a small number of studies reported external validation, while most investigations relied on internal validation approaches such as hold-out datasets or cross-validation. Calibration assessment was also infrequently reported, with only a few studies presenting metrics such as calibration curves.

Overall, 25 of the 33 evaluated studies (75.8%) were classified as having a high risk of bias, while only 6 studies (18.2%) demonstrated a low overall risk, and 2 studies (6.1%) were categorized as having an unclear risk. A detailed summary of the risk-of-bias evaluation is presented in Supplementary Table S4.

### 3.6. Meta-Analysis of ICU LOS Models

**Figure 3:**
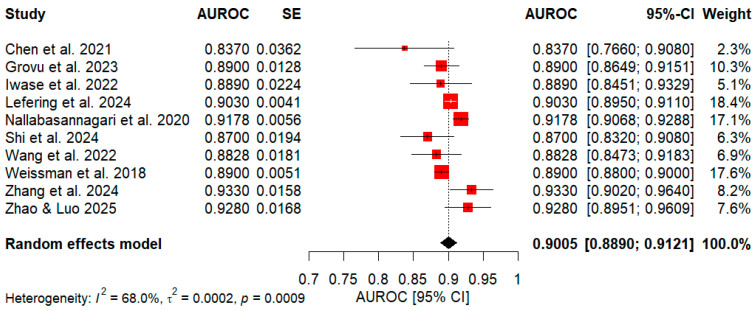
Summary of model discrimination across studies [50,53,58,60,64,68,71,72,75,76]. Weighted red squares and horizontal lines denote individual AUROCs with 95% CIs, while the bottom black diamond represents the pooled AUROC.

As depicted in Figure 3, the meta-analysis yielded an AUROC pooled effect of 0.9005 with a 95% CI: 0.8890–0.9121. Regarding performance distribution, 60.0% of the studies (6 out of 10) reported average AUROC values ≤ 0.89 (ranging from 0.83 to 0.89), whereas the remaining 40.0% (4 out of 10) reported average AUROC values ≥ 0.90 (ranging from 0.90 to 0.933). The largest weights were attributed to Lefering & Waydhas (2024) (18.4%) [60] and Weissman et al. (2018) (17.6%) [72], whereas the smallest were Chen et al. (2021) (2.3%) [50] and Iwase et al. (2022) (5.1%) [58]. The CI observed among the 10 included studies varied in width, reflecting differing levels of precision. The narrowest intervals were observed for Weissman et al. (2018) (95% CI: 0.8800–0.9000) [72] and Lefering & Waydhas (2024) (95% CI: 0.8950–0.9110) [60], indicating comparatively high precision; other relatively tight intervals included Nallabasannagaari et al. (2020) (95% CI: 0.9068–0.9288) [64]. In contrast, wider intervals indicated greater dispersion, such as Chen et al. (2021) (95% CI: 0.7661–0.9079) [50] and Shi et al. (2024) (95% CI: 0.8320–0.9080) [68]. Finally, the variance of the pooled effect was approximately $\tau^{2}$ = 0.0002 (*p* = 0.0009), and the $I^{2}$ statistic indicated moderate heterogeneity ($I^{2}$ = 68.0%).

**Figure 4:**
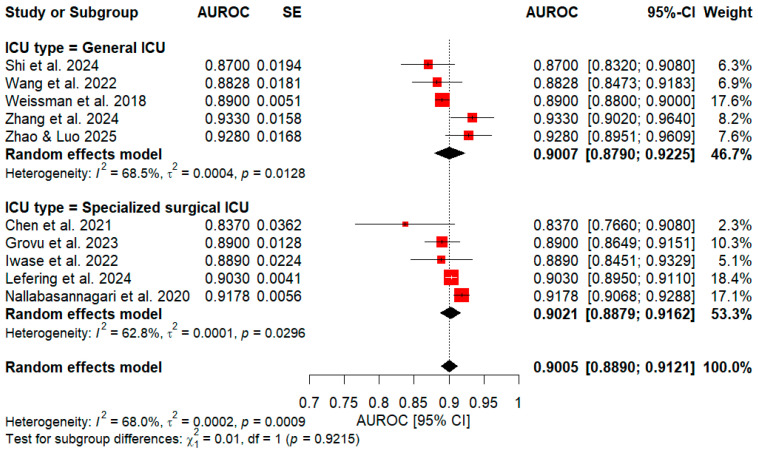
Subgroup Meta-Analysis of LOS Models by ICU Setting: Surgical vs. General [50,53,58,60,64,68,71,72,75,76]. Weighted red squares and horizontal lines denote individual AUROCs with 95% CIs, while black diamonds indicate pooled AUROCs per subgroup (specialized surgical and general ICU).

Moreover, a subgroup analysis was performed to compare specialized surgical and general ICUs, as shown in Figure 4. With respect to the specialized surgical group (5 studies), an AUROC value of 0.9021 (95% CI: 0.8879–0.9162) was obtained, with heterogeneity of $\tau^{2}$ = 0.0001 (*p* = 0.0296) and $I^{2}$ = 62.8%. Regarding the general ICU group (5 studies), the subgroup showed an AUROC of 0.9007 (95% CI: 0.8790–0.9225), with heterogeneity of $\tau^{2}$ = 0.0004 (*p* = 0.0128) and $I^{2}$ = 68.5%. Likewise, a test for subgroup differences indicated no significant distinction between the groups ($x^{2}$ = 0.01, df = 1, *p* = 0.9215).

## 4. Discussion

This systematic review synthesized current evidence on the use of ML and DL models to predict ICU LOS at admission. Across 33 eligible studies from 15 countries, we observed a rapidly expanding body of research applying data-driven models to this clinically relevant problem.

In clinical settings, mixed ICUs constituted the largest group, reflecting the heterogeneity of case mix that clinicians face daily. Major contributors were the United States and China, together accounting for 45.5% of the studies. This distribution parallels trends reported for sepsis prediction models in critical care [77], while our review also identified single studies from emerging research regions, including South Africa [46], Saudi Arabia [47], Turkey [65], and Brazil [66].

### 4.1. Meta-Analysis of ICU LOS

The meta-analysis of 10 studies demonstrated a pooled AUROC of 0.9005 (95% CI 0.8890–0.9121), indicating strong overall discrimination for identifying prolonged ICU stays. These findings suggest that contemporary ML and DL based approaches can provide reliable predictions that may support clinical decision-making and hospital resource management. When the analysis is grouped by clinical context, both the specialized-surgical and general ICU strata showed a consistent pattern. The surgical subgroup demonstrated a pooled AUROC of 0.9021, with confidence intervals tightly clustered between 0.89 and 0.92. This includes the cardiothoracic series [50], the trauma registry analysis [60], and the cardiac surgery subset [64], despite case mixes ranging from acute aortic dissection to elective colorectal resections. In parallel, five mixed medical units from three continents, including studies from diverse ICU contexts and modeling strategies, generated the same pooled discrimination, and the χ^2^ test showed no evidence of divergence between settings [56]. Across the included studies, several categories of clinical variables consistently emerged as important predictors of ICU LOS. These include vital signs (e.g., heart rate, blood pressure), laboratory values (e.g., lactate levels, creatinine, white blood cell count), demographic characteristics (e.g., age, sex), and comorbidities (e.g., cardiovascular disease, diabetes). Different studies highlighted the relevance of early physiological indicators of patient instability through feature importance maps [58] or Shapley additive explanations (SHAP) analyses [53] techniques that enhance model interpretability, which is critical for clinician trust, regulatory acceptance, and effective integration into clinical workflows. These findings suggest that admission-time clinical variables contain sufficient prognostic information to support reliable LOS prediction. Moreover, the identification of clinically interpretable predictors enhances the transparency and potential acceptance of ML-DL models in critical care settings.

The restriction of the meta-analysis to 10 of 33 studies was driven by the absence of adequate reporting of variance for AUROC in the remaining studies, rather than by thematic or qualitative selection criteria. This limitation reflects a broader reporting deficiency in the AI prediction model literature, where performance metrics are frequently reported without uncertainty measures. Importantly, the 10 included studies span four countries across three continents, multiple ICU types, diverse algorithm families, and sample sizes, suggesting that the meta-analytic subset is broadly representative of the full sample. Finally, given that 75.8% of the included studies exhibit a high risk of bias under the PROBAST+AI framework, this estimate should be interpreted as an upper bound, potentially affected by model overfitting. Consequently, rigorous external validation remains imperative for future research.

### 4.2. Modeling Approaches

Regarding modeling approaches, our findings are consistent with those of Yang et al. 2023 [77], tree-based ensemble methods—including random forests and gradient boosting algorithms—were the most commonly used techniques. This preference persisted regardless of sample size, case mix, or validation rigor. This is reflected in the structural advantage of the way they partition predictor space, making them resilient to outliers and missing values [56]. Although deep learning architectures have demonstrated comparable predictive accuracy in certain studies [57], they typically require larger datasets and greater computational resources. Consequently, the widespread adoption of ensemble methods may reflect a balance between predictive performance, interpretability, and feasibility within real-world hospital environments. This aligns with recent critical care AI frameworks, highlighting that tree-based methods are preferred for structured clinical data due to their robustness and compatibility with interpretable tools such as SHAP [78].

Methodological heterogeneity was observed among the included studies, which differed widely in validation strategies, outcome definitions, modeling approaches, and data sources. Regarding validation, most studies relied on single-split holdout datasets or cross-validation procedures within the same institution. Although such approaches involved large, well-conducted cohorts [40], only a small proportion of studies (9.9%) performed true external validation [46,71]. This limitation raises concerns regarding the generalizability of reported models, as performance may degrade when applied to new clinical settings with different patient populations or care practices. Recent comparative work indicates that ML LOS predictions often align with, but do not fully replicate, routine clinical judgment in ICU discharge decisions [79]. These findings are similar to those of Gokhale et al. [80] and Yang et al. [77], which highlighted the necessity of external validation to confirm the universality of a method for the population [77]. Thus, this scarcity reflects a broader challenge for ML and DL models to demonstrate accuracy when applied to new patient populations or healthcare systems. Expanding multi-center collaborations and promoting data sharing initiatives may help address this limitation and enhance the external validity of predictive models. Geographic concentration exacerbates this challenge, as 45.5% of the data originates from the United States and China, drawn from the MIMIC repository and individual institution records. Divergent systemic policies and clinical workflows suggest regional optimization rather than universal applicability. The review also revealed a notable imbalance in reporting model performance metrics. While AUROC was frequently reported, calibration measures were uncommon; only three studies reported calibration diagnostics [46,56,68]. This absence of calibration reporting represents a critical gap with direct clinical consequences. PROBAST+AI emphasizes that model evaluation must include both discrimination and calibration, as AUROC alone does not fully reflect clinical utility. We therefore strongly recommend that future prediction model studies in this domain report calibration metrics (calibration plots, calibration slopes, and calibration in the large) alongside discrimination.

Regarding LOS, studies differ in how it is operationalized, with thresholds ranging from more than 48 h to 14 days or longer. Such variability complicates cross-study comparisons and likely contributed to the moderate-to-high heterogeneity observed in the meta-analysis (*I^2^* = 68%). To better interpret this variability, studies can be broadly grouped into those using short-term thresholds (e.g., ≤7 days) and those using extended thresholds (e.g., >7 days). This distinction suggests that models may capture different clinical phenomena, such as early deterioration versus prolonged recovery. Such heterogeneity limits direct comparability and likely contributed to the moderate-to-high variability observed in the meta-analysis. These findings underscore the need for standardized LOS definitions or, alternatively, stratified reporting frameworks to enhance comparability across studies.

Most studies formulated the prediction task as a binary classification problem (prolonged versus non-prolonged stay) using multiple thresholds [53]. However, when such variability is pooled into a meta-analysis, it inflates between-study heterogeneity and masks how well any given model would perform once its operating point is harmonized with local discharge customs [81,82]. While the dichotomization of the LOS can facilitate clinical interpretation, it might obscure important variability in patient trajectories, reducing the granularity of the outcome and limiting the practical value of predictions. For example, expecting a LOS of six versus eight days can change ventilator allocation plans [69] or trigger an early physiotherapy consult [72]. Continuous approaches may therefore provide more informative predictions for operational planning and patient management, e.g., regression-based modeling for continuous LOS achieving a mean absolute error of under one day [61], or a temporal-pointwise convolutional network predicting remaining LOS with a median absolute deviation of 1.55 days, directly actionable for step-down planning [67]. Supporting this, a LightGBM model validated on MIMIC-III achieved an R^2^ of only 0.038 for ICU LOS, suggesting that first-day data alone capture insufficient signal due to downstream operational factors [83]. By contrast, deep networks internalize these interactions in hidden layers [64]. Nevertheless, model explanations require gradient attribution map tools that still feel foreign to many clinicians.

Although both ML and DL models demonstrated strong predictive performance, important differences were observed. Traditional ML approaches, particularly tree-based ensemble methods, were more frequently applied and showed consistent performance across diverse datasets. In contrast, DL models demonstrated comparable accuracy in some studies but were less commonly used and often required larger datasets and more complex architectures. A formal subgroup meta-analysis comparing ML and DL models was not feasible due to inconsistent reporting of performance metrics and limited availability of comparable AUROC estimates across studies. Nevertheless, the available evidence suggests that ML models remain the dominant and more practically implementable approach, while DL models represent a promising direction for capturing complex temporal patterns in ICU data.

### 4.3. Practical Implications

From a healthcare delivery perspective, unwarranted extended length of stay (LOS) increases the risk of hospital-acquired complications, morbidity, and all-cause mortality [80]. Despite the importance of having standards and targets as a reference for minimum performance levels for safety and patient flow [84], timeframes for patient throughput varied throughout regions and settings [85]. LOS in hospitals for acute care among OECD countries is 6.5 days, with Turkey (4.1 days) being the shortest and Japan the longest (16.2 days) [86]. In spite of LOS reductions depending on many factors, including patient variables, treatments, and settings [87], interventions such as lean healthcare (LH) and six sigma (SS) have shown a positive effect on reducing the average LOS, e.g., after a LH-SS intervention [88], the LOS decreased from 29 to 22 days (*p* < 0.001). Similarly, an LH intervention [89] decreased the ICU boarding time from 360.8 to 276.7 min (*p* = 0.036). Therefore, since the evidence of ICU LOS reduction through the analysis of waste and variation, improvement interventions such as LH and SS can also be complemented by simulations to provide possible scenarios without requiring the application of physical changes to an ICU process or setting [90].

From a sustainable perspective, LOS is a general measure of hospital efficiency [91] and is commonly related to cost reductions when the LOS is reduced [92]. Since ICUs account for a substantial share of hospital expenditures [1] and energy consumption, accurate prediction of ICU LOS has important implications. Reliable forecasts of ICU occupancy and patient trajectories can improve bed management, optimize staffing allocation, and reduce unnecessary delays in elective procedures. Moreover, by enabling earlier and more precise planning of critical care resources, predictive models may contribute to more efficient and sustainable healthcare delivery.

To maximize clinical utility, ICU LOS prediction models should be integrated into actionable decision-making frameworks. For example, predicted LOS can inform bed allocation strategies, optimize staffing levels, and support early discharge planning. These predictions can be incorporated into hospital decision-support systems, enabling dynamic resource allocation based on anticipated patient flow. Additionally, LOS forecasts can trigger predefined clinical pathways, such as early rehabilitation interventions or step-down unit planning for patients expected to have prolonged stays.

Despite promising predictive performance, the real-time implementation of ICU LOS prediction models presents several practical challenges. Integration with electronic health record (EHR) systems requires standardized data formats, interoperability across platforms, and the ability to process data in near real-time. Additionally, models must be seamlessly embedded into clinical workflows to provide actionable insights without increasing clinician burden. Computational constraints, data latency, and the need for continuous model updating further complicate deployment. Addressing these challenges will be essential to ensure that predictive models can function as effective real-time decision-support tools in critical care environments. Moreover, similar to related technologies such as health information systems [93], the perceived ease of use might also affect the adoption of ML and DL models.

Our findings highlight the growing role, but also the heavy reliance on publicly available clinical databases, particularly the MIMIC repository, which was used in a substantial proportion of studies. While such datasets enhance reproducibility and benchmarking, they may also limit generalizability if models capture dataset-specific patterns that do not translate to different healthcare characteristics, including regions, settings [94], and even variation in resources and healthcare expenditures [95].

Additionally, improper data partitioning in retrospective datasets may introduce risks of data leakage, particularly when temporal or patient-level dependencies are not adequately addressed. These challenges underscore the importance of rigorous validation strategies, including careful data-splitting procedures, external validation in independent cohorts, and broader geographic representation and multi-institutional validation to ensure robust, clinically applicable predictive models.

The ethical implications of AI-based prediction models must also be carefully considered. Bias may arise from imbalanced or non-representative datasets, potentially leading to disparities in model performance across patient subgroups defined by age, sex, ethnicity, or comorbidities. Ensuring fairness in predictive modeling is crucial to prevent reinforcing existing healthcare disparities. Additionally, transparency and explainability are essential for ethical deployment, enabling clinicians to understand and trust model outputs.

### 4.4. Strengths, Limitations, and Future Research

The strengths of this study include adherence to established systematic review guidelines and the incorporation of both qualitative synthesis and quantitative meta-analysis. Second, the use of the PROBAST+AI framework enabled a structured evaluation of methodological quality in machine-learning prediction studies. Finally, the review focused on models that use admission-time variables, which are particularly relevant for early triage and resource planning.

The study presents limitations that should be considered. The meta-analysis was restricted to AUROC because it was the only performance metric reported consistently across studies. This restriction limits statistical power for detecting moderator effects and may not fully capture the heterogeneity present in the wider literature. This limitation reflects a broader reporting deficiency in the AI prediction model literature, where performance metrics are frequently reported, whereas other measures, such as calibration indices, decision-curve analysis, or prediction-error metrics for continuous LOS, are rarely reported. Although hierarchical summary receiver operating characteristic models can integrate diagnostic accuracy across varying thresholds, most studies omitted the confusion matrices required for such implementations. We therefore used a generic inverse-variance random-effects approach. This reinforces the interpretation of the AUROC as a measure of average discriminatory capacity rather than a performance expectation for any single clinical threshold. Additionally, the moderate heterogeneity observed across studies reflects differences in patient populations, ICU types, modeling strategies, and outcome definitions. Consequently, the pooled estimate should be interpreted as a general benchmark rather than a universal performance expectation for any specific clinical context. Limiting inclusion to English-language publications may have excluded relevant studies published in other languages. Restricting inclusion to admission-time predictors means that our results speak most directly to early triage and resource planning; models that incorporate evolving physiological trajectories may achieve different performance profiles and deserve separate evaluation. Methodological differences in data preprocessing and missing-data handling might influence model performance, bias, and generalizability. Finally, this study was designed as a clinical prediction model assessment guided by the PRISMA 2020 and PROBAST+AI frameworks, and therefore, it did not incorporate bibliometric analyses such as keyword co-occurrence networks, journal-level metrics, or authorship productivity distributions.

Future research should prioritize several key directions. First, standardized outcome definitions and reporting guidelines would improve comparability across studies. Second, multi-center collaborations and external validation efforts are essential to assess model generalizability. Third, prospective studies evaluating the real-world clinical impact of ICU LOS prediction models are needed to determine whether these tools improve operational efficiency or patient outcomes. Fourth, transparent documentation and evaluation of preprocessing pipelines, including sensitivity analyses to assess the impact of different missing data strategies. Finally, integrating predictive models into electronic health record systems and clinical workflows will be critical for translating methodological advances into practical decision-support tools that improve healthcare delivery.

## 5. Conclusions

This systematic review and meta-analysis provide a comprehensive evaluation of machine learning and deep learning models for predicting ICU length of stay. By synthesizing the data, we found that these models exhibited strong discriminatory power, indicating that contemporary algorithms can effectively rank patients by risk of prolonged versus routine stays, thus providing support for hospital capacity planning. However, to translate this into reliable clinical support, the methodological heterogeneity, scarcity of external validation, and near absence of calibration reporting identified in this review need to be addressed.

Our subgroup meta-analysis revealed no significant difference in predictive performance between specialized surgical and general medical–surgical ICUs. This suggests that the prognostic signal derived from admission-time physiological data is consistent across varying case-mixes, reinforcing the potential for wide-scale implementation. Furthermore, the prevalence of tree-based ensemble methods, such as random forests, highlights a pragmatic balance among predictive accuracy, interpretability, and computational efficiency that is well-suited for integration into existing hospital information systems.

From a sustainability perspective, these findings underscore the potential of AI-driven prediction to enhance critical care delivery. By enabling more accurate forecasting of patient trajectories and ICU bed demand, these tools can directly contribute to reducing unnecessary resource consumption, optimizing staffing, and improving patient flow. This aligns with the core tenets of sustainable healthcare by promoting operational efficiency and system resilience without compromising the quality of care.

However, this review also identifies critical gaps that must be addressed to translate methodological advances into clinical practice. In particular, the lack of standardized ICU LOS definitions limits comparability across studies and contributes to heterogeneity in reported outcomes. Establishing common benchmarking protocols, including standardized performance metrics and validation strategies, will be essential to ensure fair and reproducible model evaluation. Furthermore, the limited number of prospective and multicenter validation studies remains a significant barrier to clinical adoption. Future research should prioritize large-scale, multi-institutional prospective studies to assess model generalizability and real-world impact. Addressing these challenges will be crucial for enabling AI-based LOS prediction models to serve as reliable and actionable tools in critical care settings.

## Figures and Tables

**Table 1:** Characteristics of the included studies.

Author and Year	Country and Clinical Setting	Data Source, Study Period, and Design	Sample Size; Inclusion Criteria; and Case Mix	Outcome
Achilonu et al. 2021 [46]	South Africa; Surgical oncology ward	CRCSA; 2015–2019; retrospective	*n* = 383; CRC surgery	Hospital LOS, binary (LOS ≤ 9 d, LOS > 9 d)
Alabbad et al. 2022 [47]	Saudi Arabia; General ICU	King Fahad Univ. Hosp.; NR; retrospective	*n* = 895; COVID-19 ICU	ICU LOS, 9 bins (ordinal)
Alsinglawi et al. 2020 [7]	Australia; Mixed adult ICU	MIMIC-III; 2001–2012; retrospective	*n* = 1592; heart failure	ICU LOS (continuous, d)
Alsinglawi et al. 2022 [48]	Australia; Mixed adult ICU	MIMIC-III; 2001–2012; retrospective	*n* = 52,423; lung cancer	ICU LOS, binary (LOS ≤ 7 d, LOS > 7 d)
Batista and Sanchez 2020 [49]	United States; Mixed adult ICU	MIMIC-III; 2001–2012; retrospective	*n* = 61,293; ≥50 years and respiratory disease	ICU LOS, 3 bins (LOS ≤ 3 d, 3 d < LOS < 5 d, LOS ≥ 5 d)
Chen et al. 2021 [50]	China; Cardiothoracic Surgery ICU	Guangdong Cardiovascular Inst.; 2016–2019; retrospective	*n* = 353; type-A aortic dissection surgery	ICU LOS, 4 bins (<4 d, 4–7 d, 7–10 d, and >10 d)
Chrusciel et al. 2021 [51]	France; ED & wards	Dr Warehouse; 2019; observational retrospective cohort study	*n* = 5006; ED ≥ 2 d	Hospital LOS, binary (<7 d, ≥7 d)
Daghistani et al. 2019 [52]	Saudi Arabia; Cardiac ward ± ICU	KACC; 2008–2016; retrospective	*n* = 16,414; cardiology	Hospital LOS, 3 bins (<3 d, 3–5 d, >5 d)
Grovu et al. 2023 [53]	United States; General ward	National inpatient sample database; 2016–2018; cross-sectional, retrospective	*n* = 5831; lupus flare	Hospital LOS, binary, above or below 7 d, 8 d, and 14 d
Guo et al. 2025 [54]	China; Mixed adult ICU	MIMIC-IV; 2008–2019; retrospective	*n* = 2374; atherosclerotic cardiovascular disease	Prolonged hospital LOS and ICU LOS
Hasan et al. 2023 [55]	United States; Mixed adult ICU	MIMIC-III demo; 2001–2012; retrospective	*n* = 48; complete features	ICU LOS (continuous, d)
Hempel et al. 2023 [56]	Germany; Mixed adult ICU	MIMIC-IV; 2008–2019; retrospective	*n* = 41,473; adults	ICU LOS, binary (LOS < 4 d, LOS ≥ 4 d)
Hu et al. 2022 [57]	United States; Mixed adult ICU	MIMIC-III; 2001–2012; retrospective	*n* = 894; diabetes ICU	ICU LOS, binary (LOS < 10 d, LOS ≥ 10 d)
Iwase et al. 2022 [58]	Japan; Mixed adult ICU	Chiba University EMR; 2008–2019; retrospective	*n* = 12,747; consecutive ICU	ICU LOS, 3 bins (LOS < 1 w, 1 w ≤ LOS ≤ 2 w, LOS > 2 w)
LaFaro et al. 2015 [59]	United States; Cardiac surgery ICU	Westchester Medical Center; NR; retrospective	*n* = 185; Cardiac surgery	ICU LOS (continuous, h)
Lefering & Waydhas 2024 [60]	Germany; Trauma ICU	Trauma Register DGU; 2014–2018; retrospective	*n* = 180,240; trauma survivors	ICU LOS, binary (<8 d, ≥8 d)
Li et al. 2019 [61]	China; Mixed adult ICU	Sichuan People’s Hospital EHR; 2015–2018; retrospective	*n* = 1214; unplanned ICU	ICU LOS (continuous, d)
Mekhaldi et al. 2021 [62]	France; General wards	Microsoft open dataset; NR; retrospective	*n* = 100,000; non-ICU stays	Hospital LOS (continuous, d)
Mollaei et al. 2021 [63]	Portugal; Cardiothoracic ICU	Lisbon surgical dataset; 2011–2020; retrospective	*n* = 7364; Cardiothoracic surgery	ICU LOS, binary (LOS ≤ 2 d, LOS > 2 d)
Nallabasannagaari et al. 2020 [64]	United States; Mixed adult ICU	MIMIC-III; 2001–2012; retrospective	*n* = 42,818; first ICU ≥ 24 h	ICU LOS, binary (LOS < 7 d, LOS ≥ 7 d)
Özbilen et al. 2023 [65]	Turkey; General wards	Ordu Univ. HIMS; 2020–2021; retrospective and observational	*n* = 118; COVID-19 adults	Hospital LOS, binary (LOS ≤ 5, LOS > 5 d)
Peres et al. 2022 [66]	Brazil; Mixed adult ICU	Epimed Monitor; 2019; retrospective	*n* = 99,492; ICU > 6 h	ICU LOS (continuous, days)
Rocheteau et al. 2021 [67]	United Kingdom; Mixed adult ICU	eICU + MIMIC-IV; 2014–2019; retrospective	*n* = 146,671 + 69,609; ICU ≥ 5 h	ICU LOS (continuous, h)
Shi et al. 2024 [68]	China; Mixed adult ICU	MIMIC-IV; 2008–2019; retrospective	*n* = 669; Diabetic ketoacidosis	ICU LOS, binary (LOS < 75 h, LOS ≥ 75 h)
Stieger et al. 2025 [69]	Switzerland and South Korea; Post-op adult ICU	VitalDB; 2016–2017; retrospective	*n* = 6043; general anesthesia for non-cardiac surgery	ICU LOS, binary, from ≥1 d up to ≥7 d
Tanutsiriteeradet et al. 2024 [70]	Thailand; Hospital ICU	MIMIC-III; simulation	*n* = 42,692; adult ICU	ICU LOS, 4 bins (LOS < 3 d, 3 ≤ LOS ≤ 7 d, 7 d < LOS < 14 d)
Tella and Balasundaram 2025 [4]	India; Mixed adult ICU	MIMIC-III; 2001–2012; retrospective	*n* = 40,000; adult ICU	ICU LOS (continuous, d)
Wang et al. 2022 [71]	China; Cardiac surgery ICU	Wuhan Union Hospital EHR; 2017–2020; retrospective	*n* = 365; heart-transplant ICU	ICU LOS, binary (LOS ≤ 9.08 d, LOS > 9.08 d)
Weissman et al. 2018 [72]	United States; Mixed adult ICU	MIMIC-III; 2001–2012; retrospective	*n* = 25,947; ICU ≥ 48 h	ICU LOS, binary (LOS < 7 d, LOS ≥ 7 d)
Zebin et al. 2019 [73]	United Kingdom; Mixed adult ICU	MIMIC-III; 2001–2012; retrospective	*n* = 26,800; ICU ≥ 24 h	Hospital LOS, binary (LOS ≤ 7 d, LOS > 7 d)
Zhang and Kuo 2024 [74]	United States; Mixed adult ICU	MIMIC-IV; 2008–2019; retrospective	*n* = 18,572; two admissions	ICU LOS, binary (LOS < 3 d, LOS ≥ 3 d)
Zhang et al. 2024 [75]	China; Vascular surgery ICU	Guangxi Medical University EHR; 2012–2021; retrospective	*n* = 266; post-Endovascular aneurysm repair	ICU LOS (continuous, h)
Zhao and Luo 2025 [76]	China; Neurosurgical ICU	Jingzhou First People’s Hospital EHR; 2022–2023; retrospective	*n* = 325; aneurysm embolization	Hospital LOS, binary (LOS < 13 d, LOS ≥ 13 d)

Note: CRCSA: Colorectal cancer surgical audit; CRC: Colorectal cancer; d: Days; NR: Not reported; ED: Emergency department; EMR: Electronic medical record; w: Week; DGU: German Trauma Society registry; HIMS: Hospital information management system; eICU: eICU Collaborative Research Database.

**Table 2:** Model development and performance metrics.

Author and Year	Best Performance Model	Validation Strategy (Training-Testing)	Data Preprocessing	AUROC [95% CI]	Additional Key Metrics
Achilonu et al. 2021 [46]	Logistic Regression	MC CV	LASSO; Random-forest imputation	0.82 [0.80–0.85]	Sensitivity = 0.78, Specificity = 0.71, Accuracy = 0.79
Alabbad et al. 2022 [47]	Random Forest	Holdout (80/20) with 3-fold cross-validation	Boruta; kNNimp (k = 3–15); SMOTE	NR	Accuracy = 0.94, Precision = 0.94, Recall = 0.94, F1 score = 0.94
Alsinglawi et al. 2020 [7]	Gradient Boosting Regression	(64/34)	Correlation; Impute missing values	NR	R^2^ = 0.84 ± 0.07, MAE = 2.00 d
Alsinglawi et al. 2022 [48]	Random Forest + ADASYN	CV KF10 + H	CS and RFE; MDHDI; ADASYN	0.98 [0.953–1]	Sensitivity = 1.00, Specificity = 1.00
Batista and Sanchez 2020 [49]	Random Forest	NR	EXP	NR	Accuracy = 0.603, Cohen’s Kappa = 0.203
Chen et al. 2021 [50]	Random Forest	(70/30) + KF5	MICE (10×); Kendall correlation	0.84 [0.77–0.91]	NR
Chrusciel et al. 2021 [51]	Random Forest	(80/20)	SRF (UMLS concepts) + one-hot encoding + affirmation filter	NR	Accuracy = 0.75; F1 score = 0.76; Recall = 0.77
Daghistani et al. 2019 [52]	Random Forest	KF10	Ranker search of Weka software	0.94	Accuracy = 0.80; F1 score = 0.80
Grovu et al. 2023 [53]	Extreme Gradient Boosting	KF10	Recursive feature elimination with CV	0.89 [0.88–0.93]	Accuracy = 0.95, F1 score = 0.56
Guo et al. 2025 [54]	Logistic Regression	(80/20)	LASSO and Boruta; Drop25% + MICE	0.832	Sensitivity = 0.80, Specificity = 0.72, Accuracy = 0.74, F1 score = 0.74
	Light Gradient Boosting			0.740	Sensitivity = 0.508, Specificity = 0.88, Accuracy = 0.68, F1 score = 0.67
Hasan et al. 2023 [55]	XGBoost Regressor	(80/20)	EXP; CC	NR	R^2^ = 0.86, RMSE = 1.20 d
Hempel et al. 2023 [56]	Random Forest	(80/20) × 10	CC	0.80	Accuracy = 0.81; F1 score = 0.44
Hu et al. 2022 [57]	Neural Network	(90/10) + KF10	MB; one-hot encoding	NR	R^2^ = 0.40, MAE = 1.94 d
Iwase et al. 2022 [58]	Random Forest	(80/20)	DQ; IMV (10×)	0.89 [0.85–0.94]	Accuracy = 0.83
LaFaro et al. 2015 [59]	Neural Network	(90/10)	MB; CC	NR	R^2^ = 0.41
Lefering & Waydhas 2024 [60]	Logistic Regression	(60/40)	MB	0.90 [0.90–0.91]	r = 0.61
Li et al. 2019 [61]	LASSO	(70/30) + KF10	MB; Mode/zero + Drop > 74%; all admitted patients	NR	MAE = 0.87 d; R^2^ = 0.35
Mekhaldi et al. 2021 [62]	Gradient Boosting	(70/30)	SMOTE; one-hot encoding	NR	MAE = 0.44 d; R^2^ = 0.94
Mollaei et al. 2021 [63]	Random Forest	H80/20	NR; Mean/Mode	NR	Accuracy = 0.76
Nallabasannagaari et al. 2020 [64]	Deep learning model	(85/15)	NaN-token	0.88 [0.87–0.89]	F1 score = 0.61, PR-AUC = 0.68
Özbilen et al. 2023 [65]	k-nearest neighbors	H80/20 + KF10	NR; CC	NR	Accuracy = 0.92 [0.73–0.99]; F1 score = 0.89
Peres et al. 2022 [66]	Stacked Random Forest + Logistic Regression	(80/20) + External validation cohort	DQ; Drop > 30% + MICE	NR	RMSE = 3.82 d; MAE = 2.52 d
Rocheteau et al. 2021 [67]	Time-Partitioned Convolutional Neural Network	(70/15/15)	Forward-fill + decay-flags	NR	MAD = 2.28 d; R^2^ = 0.46
Shi et al. 2024 [68]	Logistic Regression nomogram	(70/30)	LASSO; MB; MICE < 20% + Excl > 20%	0.86 [0.80–0.92]	Hosmer–Lemeshow test *p*-value = 0.37
Stieger et al. 2025 [69]	Stacked learner + Logistic Regression + Random Forest	(60/40) + 2 × 2KF	DQ; Drop ≥ 66%	0.93 [0.92–0.94]	PR-AUC = 0.78
Tanutsiriteeradet et al. 2024 [70]	Transformer Deep neural network	(75/25) + KF5	Interpolation for missing data	NR	Accuracy = 0.82, Precision = 0.82
Tella and Balasundaram 2025 [4]	Stacked Random Forest + SVM + k-nearest neighbors	(70/30)	EXP; Mean/Med/Mode + miss-flags	NR	MAE = 1.78 d, R^2^ = 0.86
Wang et al. 2022 [71]	Extreme Gradient Boosting	(70/30)	LASSO; MB; Median-imputation	0.88 [0.86–0.93]	Accuracy = 0.87, Sensitivity = 0.98, Specificity = 0.51
Weissman et al. 2018 [72]	Gradient Boosting	(75/25) + 5 × KF10	EXP	0.89 [0.88–0.90]	NR
Zebin et al. 2019 [73]	Autoencoder + Deep neural network	(80/10/10)	NR; Outliers removed	NR	Accuracy = 0.78, Precision = 0.80, Recall = 0.78
Zhang and Kuo 2024 [74]	Random Forest	(50/25/25) + KF10	NR	0.72 [0.71–0.73]	F1 score = 0.74, Sensitivity = 0.80
Zhang et al. 2024 [75]	Logistic Regression nomogram	Internal	HYB	0.93 [0.90–0.96]	Sensitivity = 0.795, Specificity = 0.495, Precision = 0.683, F1 score = 0.735
Zhao and Luo 2025 [76]	Random Forest	(70/30)	HYB; Drop > 20% + Median-imputation	0.93 [0.90–0.96]	Sensitivity = 0.82, Specificity = 0.84, Accuracy = 0.84, F1 score = 0.69

Note: MC CV: Monte Carlo cross-validation; LASSO: Least Absolute Shrinkage and Selection Operator; SMOTE: Synthetic Minority Over-Sampling Technique; NR: not reported; R^2^: coefficient of determination; MAE: mean absolute error; CV: cross-validation; KF10: 10-fold cross-validation; KF5: 5-fold cross-validation; CS: correlation screening; RFE: recursive feature elimination; MDHDI: Missing-data handling via deletion/imputation; EXP: expert-defined feature; MICE: multiple imputation by chained equations; SRF: semantic rule-based filter; UMLS: Unified Medical Language System; HYB: hybrid pipeline (combined filters/wrappers/steps); CC: complete-case analysis; RMSE: root mean square error; MB: model-based approach; DQ: data-quality filtering; r: Pearson correlation coefficient; NaN-token: missing-value token; PR-AUC: precision–recall AUC; decay-flags: time-decay indicator flags; MAD: median absolute deviation; miss-flags: missingness indicator flags; 5 × KF10: five repeats of 10-fold cross-validation.

## Data Availability

Data used in this review are included in the Supplementary Materials.

## Supplementary Materials

The following supporting information can be downloaded at: https://www.mdpi.com/article/10.3390/healthcare14091131/s1, Table S1: PRISMA checklist; Table S2: PICOTS framework; Table S3: Search strategy and PICOTS-based eligibility criteria; Table S4: Risk of bias assessment [4,7,46,47,48,49,50,51,52,53,54,55,56,57,58,59,60,61,62,63,64,65,66,67,68,69,70,71,72,73,74,75,76]; Table S5: Meta-analysis dataset (AUROC) [50,53,58,60,64,68,71,72,75,76].

## Abbreviations

The following abbreviations are used in this manuscript:

AI	Artificial intelligence
AUROC	Area under the receiver operating characteristic curve
CI	Confidence interval
DL	Deep learning
EHR	Electronic health record
GB	Gradient boosting methods
ICU	Intensive care unit
LOS	Length of stay
MIMIC	Medical Information Mart for Intensive Care
ML	Machine learning
PRISMA	Preferred reporting items for systematic reviews and meta-analyses
RF	Random forests
RoB	Risk of bias
SVM	Support vector machines
