# Supplementary material for: Evaluating Artificial Intelligence Models for ICU Length of Stay Prediction: A Systematic Review and Meta-Analysis
Source: Healthcare (Basel). 2026 Apr 23;14(9):1131. doi: 10.3390/healthcare14091131 (PMC13163238; doi:10.3390/healthcare14091131)
Supplement: Supplementary file 1 [file healthcare-14-01131-s001.zip › healthcare-4235347-supplementary.pdf]

Supplementary Table S1. PRISMA Checklist.

Section and Topic	Item #	Checklist item	Location where item is reported
**TITLE**			
Title	1	Identify the report as a systematic review.	p. 1
**ABSTRACT**			
Abstract	2	See the PRISMA 2020 for Abstracts checklist.	p. 1
**INTRODUCTION**			
Rationale	3	Describe the rationale for the review in the context of existing knowledge.	p. 2–3 (Introduction)
Objectives	4	Provide an explicit statement of the objective(s) or question(s) the review addresses.	p. 2–3 (End of Introduction)
**METHODS**			
Eligibility criteria	5	Specify the inclusion and exclusion criteria for the review and how studies were grouped for the syntheses.	p. 3 (Eligibility criteria)
Information sources	6	Specify all databases, registers, websites, organisations, reference lists and other sources searched or consulted to identify studies. Specify the date when each source was last searched or consulted.	p. 3 (Information Sources and Search Strategy)
Search strategy	7	Present the full search strategies for all databases, registers and websites, including any filters and limits used.	p. 3 (Information Sources and Search Strategy; Supplementary Tables 2–3)
Selection process	8	Specify the methods used to decide whether a study met the inclusion criteria of the review, including how many reviewers screened each record and each report retrieved, whether they worked independently, and if applicable, details of automation tools used in the process.	p. 3–4 (Study selection and data extraction)
Data collection process	9	Specify the methods used to collect data from reports, including how many reviewers collected data from each report, whether they worked independently, any processes for obtaining or confirming data from study investigators, and if applicable, details of automation tools used in the process.	p. 4 (Study selection and data extraction)
Data items	10a	List and define all outcomes for which data were sought. Specify whether all results that were compatible with each outcome domain in each study were sought (e.g. for all measures, time points, analyses), and if not, the methods used to decide which results to collect.	p. 4 (Study selection and data extraction)
	10b	List and define all other variables for which data were sought (e.g. participant and intervention characteristics, funding sources). Describe any assumptions made about any missing or unclear information.	p. 4 (Study selection and data extraction)
Study risk of bias assessment	11	Specify the methods used to assess risk of bias in the included studies, including details of the tool(s) used, how many reviewers assessed each study and whether they worked independently, and if applicable, details of automation tools used in the process.	p. 4 (Quality assessment; PROBAST+AI; Supplementary Table 4)
Effect measures	12	Specify for each outcome the effect measure(s) (e.g. risk ratio, mean difference) used in the synthesis or presentation of results.	p. 4 (Data synthesis)
Synthesis methods	13a	Describe the processes used to decide which studies were eligible for each synthesis (e.g. tabulating the study intervention characteristics and comparing against the planned groups for each synthesis (item #5)).	p. 4 (Data synthesis)
	13b	Describe any methods required to prepare the data for presentation or synthesis, such as handling of missing summary statistics, or data conversions.	p. 4 (Data synthesis)
	13c	Describe any methods used to tabulate or visually display results of individual studies and syntheses.	p. 4 (Data synthesis, forest plot, Table 1,

Section and Topic	Item #	Checklist item	Location where item is reported
			Table 2 and Figure 5 subgroup)
	13d	Describe any methods used to synthesize results and provide a rationale for the choice(s). If meta-analysis was performed, describe the model(s), method(s) to identify the presence and extent of statistical heterogeneity, and software package(s) used.	p. 4 (Data synthesis)
	13e	Describe any methods used to explore possible causes of heterogeneity among study results (e.g. subgroup analysis, meta-regression).	p. 4 (Data synthesis)
	13f	Describe any sensitivity analyses conducted to assess robustness of the synthesized results.	NR
Reporting bias assessment	14	Describe any methods used to assess risk of bias due to missing results in a synthesis (arising from reporting biases).	NR
Certainty assessment	15	Describe any methods used to assess certainty (or confidence) in the body of evidence for an outcome.	NR
**RESULTS**			
Study selection	16a	Describe the results of the search and selection process, from the number of records identified in the search to the number of studies included in the review, ideally using a flow diagram.	p. 4–5 (Study selection; Figure 1 PRISMA flow diagram)
	16b	Cite studies that might appear to meet the inclusion criteria, but which were excluded, and explain why they were excluded.	NR
Study characteristics	17	Cite each included study and present its characteristics.	p. 6–8 (Study characteristics; Table 1)
Risk of bias in studies	18	Present assessments of risk of bias for each included study.	p. 13 (Risk of Bias; Supplementary Table 4)
Results of individual studies	19	For all outcomes, present, for each study: (a) summary statistics for each group (where appropriate) and (b) an effect estimate and its precision (e.g. confidence/credible interval), ideally using structured tables or plots.	p. 9–11 (Section 3.3; Table 2)
Results of syntheses	20a	For each synthesis, briefly summarise the characteristics and risk of bias among contributing studies.	p. 13–14 (Section 3.5 Meta-analysis)
	20b	Present results of all statistical syntheses conducted. If meta-analysis was done, present for each the summary estimate and its precision (e.g. confidence/credible interval) and measures of statistical heterogeneity. If comparing groups, describe the direction of the effect.	p. 13–14 (Section 3.5; Figure 4)
	20c	Present results of all investigations of possible causes of heterogeneity among study results.	p. 14 (Section 3.5; Figure 5)
	20d	Present results of all sensitivity analyses conducted to assess the robustness of the synthesized results.	NR
Reporting biases	21	Present assessments of risk of bias due to missing results (arising from reporting biases) for each synthesis assessed.	NR
Certainty of evidence	22	Present assessments of certainty (or confidence) in the body of evidence for each outcome assessed.	NR
**DISCUSSION**			
Discussion	23a	Provide a general interpretation of the results in the context of other evidence.	p. 14–17 (Discussion)
	23b	Discuss any limitations of the evidence included in the review.	p. 17 (Limitations subsection)
	23c	Discuss any limitations of the review processes used.	p. 17 (Limitations subsection)

Section and Topic	Item #	Checklist item	Location where item is reported
	23d	Discuss implications of the results for practice, policy, and future research.	p. 17–18 (End of Discussion and Section 5 Conclusion)
**OTHER INFORMATION**			
Registration and protocol	24a	Provide registration information for the review, including register name and registration number, or state that the review was not registered.	p. 3 (Section 2; PROSPERO CRD420251089785)
	24b	Indicate where the review protocol can be accessed, or state that a protocol was not prepared.	p. 3 (Section 2; PROSPERO CRD420251089785
	24c	Describe and explain any amendments to information provided at registration or in the protocol.	NR
Support	25	Describe sources of financial or non-financial support for the review, and the role of the funders or sponsors in the review.	p. 18
Competing interests	26	Declare any competing interests of review authors.	p. 19
Availability of data, code and other materials	27	Report which of the following are publicly available and where they can be found: template data collection forms; data extracted from included studies; data used for all analyses; analytic code; any other materials used in the review.	p. 19

Supplementary Table S2. PICOTS Framework.

Item	Explanation
**P**opulation	Adult patients (≥18 years) in critical care inpatient cohorts, primarily ICU populations (general, cardiac, trauma, surgical, and mixed medical–surgical ICUs). Adult cohorts (e.g., sepsis, post-operative) were eligible when the target outcome was a length of stay (LOS). Pediatric, neonatal, and obstetric populations were excluded.
**I**ndex model(s)	Prediction models developed and/or evaluated using machine learning (ML) or deep learning (DL) techniques for LOS prediction, using structured and/or unstructured data. Both regression and classification formulations were eligible (e.g., random forest, gradient boosting, DNN/CNN/RNN).
**C**omparator model(s)	No predefined comparator was required. When reported, comparisons against baseline/statistical models or clinical scores were extracted descriptively.
**O**utcome(s)	LOS outcomes as reported by the primary studies, including ICU LOS and, where applicable in the included corpus, hospital LOS. LOS was considered either as a continuous measure (e.g., days) or as threshold category (e.g., prolonged LOS such as ≥3 days).
**T**iming	Admission time or early prediction using data available at or near admission to the relevant setting. Models requiring post-discharge data were excluded.
**S**etting and intended use of the prediction model	Adult inpatient ICUs and selected acute care inpatient environments represented in the included studies. Intended use: support early prognostic assessment and operational/resource planning (e.g., bed management) in critical care settings.

Supplementary Table S3. Search Strategy and PICOTS-Based Eligibility Criteria

i. *Predictive Modeling of ICU Length of Stay*

Database	Search String	Results
PubMed / MEDLINE	("length of stay"[Title/Abstract] OR LOS[Title/Abstract]) AND ("intensive care"[Title/Abstract] OR ICU[Title/Abstract]) AND ("predictive model"[Title/Abstract] OR "machine learning"[Title/Abstract] OR "deep learning"[Title/Abstract] OR "artificial intelligence"[Title/Abstract] OR algorithm*[Title/Abstract]) NOT (pediatric [Title/Abstract] OR animal[Title/Abstract])	585
Web of Science	TS= ("length of stay") AND TS=("intensive care" OR ICU) AND TS=("predictive model" OR prediction OR "machine learning" OR "deep learning" OR algorithm) NOT TS=(pediatric OR animal OR mortality OR readmission)	385
EBSCO (Host CINAHL)	TI ("length of stay") AND (TX ("intensive care" OR ICU)) AND (TX ("predictive model" OR prediction OR "machine learning" OR "deep learning" OR algorithm*)) AND NOT (TX (pediatric OR animal OR mortality OR readmission))	272
Elsevier / ScienceDirect	"length of stay" ICU ("machine learning" OR "deep learning") prediction -pediatric -mortality -readmission	94
Emerald Insight	"length of stay" AND ("intensive care" OR ICU) AND ("predictive model" OR prediction OR "machine learning" OR "deep learning" OR algorithm) AND NOT (pediatric OR animal OR mortality OR readmission)	90
IEEE Xplore	("length of stay") AND ("intensive care" OR ICU) AND ("predictive model" OR prediction OR "machine learning" OR "deep learning" OR algorithm) NOT (pediatric OR animal OR mortality OR readmission)	87
Springer Nature Link	"length of stay" AND ("intensive care" OR ICU) AND ("predictive model" OR prediction OR "machine learning" OR "deep learning" OR algorithm) AND NOT (pediatric OR animal OR mortality OR readmission)	957
Google Scholar	"length of stay" AND ("intensive care" OR ICU) AND ("machine learning" OR "deep learning") AND ("predictive model" OR prediction) -pediatric -animal -mortality -readmission	1 140

ii. Validation and Performance Metrics of LOS Prediction Models

Database	Search String	Results
PubMed / MEDLINE	("length of stay"[Title/Abstract] OR LOS[Title/Abstract]) AND<br>("intensive care"[Title/Abstract] OR ICU[Title/Abstract]) AND<br>("machine learning"[Title/Abstract] OR "deep learning"[Title/Abstract] OR "predictive model"[Title/Abstract]) AND<br>("validation"[Title/Abstract] OR "external validation"[Title/Abstract] OR AUROC[Title/Abstract] OR "model performance"[Title/Abstract] OR calibration[Title/Abstract] OR discrimination[Title/Abstract])<br>NOT (pediatric[Title/Abstract] OR animal[Title/Abstract] OR mortality[Title/Abstract] OR readmission[Title/Abstract])	44
Web of Science	TS=("length of stay") AND<br>TS=("intensive care" OR ICU) AND<br>TS=("predictive model" OR "machine learning" OR "deep learning") AND<br>TS=("validation" OR "external validation" OR AUROC OR "model performance" OR calibration OR discrimination) NOT<br>TS=(pediatric OR animal OR mortality OR readmission)	35
EBSCO (Host CINAHL)	TI ("length of stay") AND<br>TX ("intensive care" OR ICU) AND<br>TX ("predictive model" OR "machine learning" OR "deep learning") AND<br>TX ("validation" OR "external validation" OR AUROC OR "model performance" OR calibration OR discrimination) AND NOT<br>TX (pediatric OR animal OR mortality OR readmission)	263

Elsevier / ScienceDirect	"length of stay" ICU ("machine learning" OR "deep learning") ("validation" OR AUROC OR "model performance" OR calibration OR discrimination) -pediatric -mortality -readmission	104
Emerald Insight	"length of stay" AND ("intensive care" OR ICU) AND ("predictive model" OR "machine learning" OR "deep learning") AND ("validation" OR AUROC OR "model performance" OR calibration OR discrimination) AND NOT (pediatric OR animal OR mortality OR readmission)	83
IEEE Xplore	("length of stay") AND ("intensive care" OR ICU) AND ("machine learning" OR "deep learning" OR "predictive model") AND ("validation" OR AUROC OR calibration OR discrimination) NOT (pediatric OR animal OR mortality OR readmission)	10
Springer Nature Link	"length of stay" AND ("intensive care" OR ICU) AND ("predictive model" OR "machine learning" OR "deep learning") AND ("validation" OR AUROC OR "model performance" OR calibration OR discrimination) AND NOT (pediatric OR animal OR mortality OR readmission)	854
Google Scholar	"length of stay prediction" AND ("intensive care" OR ICU) AND ("validation" OR AUROC OR "model performance" OR calibration OR discrimination) AND ("machine learning" OR "deep learning") -pediatric -mortality -readmission	75

iii. Clinical Databases Driving LOS Predictive Modeling Research

Database	Search String	Results
PubMed / MEDLINE	("length of stay"[Title/Abstract] OR LOS[Title/Abstract]) AND ("intensive care"[Title/Abstract] OR ICU[Title/Abstract]) AND ("MIMIC"[Title/Abstract] OR "eICU"[Title/Abstract] OR "electronic health record"[Title/Abstract] OR EHR[Title/Abstract] OR "clinical database"[Title/Abstract] OR "registry"[Title/Abstract]) AND ("machine learning"[Title/Abstract] OR "deep learning"[Title/Abstract]) NOT (pediatric[Title/Abstract] OR animal[Title/Abstract] OR mortality[Title/Abstract] OR readmission[Title/Abstract])	18
Web of Science	TS=("length of stay") AND TS=("intensive care" OR ICU) AND TS=("MIMIC" OR "eICU" OR "electronic health record" OR EHR OR "clinical database" OR registry) AND TS=("machine learning" OR "deep learning") NOT TS=(pediatric OR animal OR mortality OR readmission)	20
EBSCO (Host CINAHL)	TI ("length of stay") AND TX ("intensive care" OR ICU) AND TX ("MIMIC" OR "eICU" OR "electronic health record" OR EHR OR "clinical database" OR registry) AND TX ("machine learning" OR "deep learning") AND NOT TX (pediatric OR animal OR mortality OR readmission)	242
Elsevier / ScienceDirect	"length of stay" ICU ("MIMIC" OR "eICU" OR "electronic health record" OR EHR OR "clinical database" OR registry) ("machine learning" OR "deep learning") -pediatric -mortality -readmission	66
Emerald Insight	"length of stay" AND ("intensive care" OR ICU) AND ("MIMIC" OR "eICU" OR "electronic health record" OR EHR OR "clinical database" OR registry) AND ("machine learning" OR "deep learning") AND NOT (pediatric OR animal OR mortality OR readmission)	10
IEEE Xplore	("length of stay") AND ("intensive care" OR ICU) AND ("MIMIC" OR "eICU" OR "EHR" OR "electronic health record" OR "clinical database" OR registry) AND ("machine learning" OR "deep learning") NOT (pediatric OR animal OR mortality OR readmission)	24
Springer Nature Link	"length of stay" AND ("intensive care" OR ICU) AND ("MIMIC" OR "eICU" OR "electronic health record" OR EHR OR "clinical database" OR registry) AND ("machine learning" OR "deep learning") AND NOT (pediatric OR animal OR mortality OR readmission)	65
Google Scholar	"length of stay" AND ("intensive care" OR ICU) AND ("MIMIC" OR "eICU" OR "electronic health record" OR EHR OR "clinical database" OR registry) AND ("machine learning" OR "deep learning") -pediatric -mortality -readmission	782

Supplementary Table S4. Risk of Bias Assessment.

Author (Year)	D1 RoB	D2 RoB	D3 RoB	D4 RoB	A1 Applic	A2 Applic	A3 Applic	Overall RoB	Overall Applic
Achilonu et al. (2021) [46]	Low	High	High	Low	Low	High	Low	High	High
Alabbad et al. (2022) [47]	Low	Low	Low	High	Low	Low	Low	High	Low
Alsinglawi et al. (2020) [7]	Low	Low	Low	High	Low	Low	Low	High	Low
Alsinglawi et al. (2022) [48]	Unclear	Unclear	Unclear	High	High	Unclear	Low	High	High
Batista & Sanchez-Arias (2020) [49]	High	Unclear	Unclear	High	High	Unclear	Low	High	High
Chen et al. (2021) [50]	Low	Low	Low	High	Low	Low	Low	High	Low
Chrusciel et al. (2021) [51]	High	Low	Low	High	Low	Low	Low	High	Low
Daghistani et al. (2019) [52]	Low	Low	Low	High	Low	Low	Low	High	Low
Grovu et al. (2023) [53]	Low	High	High	Low	Low	High	Low	High	High
Guo et al. (2025) [54]	Low	Low	Low	High	Low	Low	Low	High	Low
Hasan et al. (2023) [55]	High	Low	Low	High	Low	Low	Low	High	Low
Hempel et al. (2023) [56]	High	Low	Low	High	Low	Low	Low	High	Low
Hu et al. (2022) [57]	High	Low	Low	High	Low	Low	Low	High	Low
Iwase et al. (2022) [58]	High	Low	Low	Low	Low	Low	Low	High	Low
LaFaro et al. (2015) [59]	Low	Low	Low	High	Low	Low	Low	High	Low
Lefering & Waydhas (2024) [60]	High	Low	Low	High	Low	Low	Low	High	Low
Li et al. (2019) [61]	Low	Low	Low	Low	Low	Low	Low	Low	Low
Mekhaldi et al. (2021) [62]	Unclear	Unclear	Unclear	High	Unclear	Unclear	Low	High	Unclear
Mollaei et al. (2021) [63]	Low	Low	Low	High	Low	Low	Low	High	Low
Nallabasannagari et al. (2020) [64]	High	Unclear	Unclear	High	High	High	Low	High	High
Özbilen et al. (2023) [65]	High	Low	Low	High	Low	Low	Low	High	Low
Peres et al. (2022) [66]	Low	Unclear	Low	Unclear	Low	High	Low	Low	Low
Rocheteau et al. 2021 [67]	Low	Low	High	High	Low	Low	Low	High	Low
Shi et al. (2024) [68]	High	Unclear	Unclear	High	High	Low	High	High	High
Stieger et al. (2025) [69]	Low	Low	Low	Low	Low	Low	Low	Low	Low
Tanutsiriteeradet et al. (2024) [70]	Low	Low	Low	Low	Low	Low	Low	Low	Low
Tella & Balasundaram (2025) [4]	Low	Low	Low	Low	Low	Low	Low	Low	Low
Wang et al. (2022) [71]	Unclear	Unclear	High	High	High	High	High	High	High
Weissman et al. (2018) [72]	Low	Low	Low	Low	Low	Low	Low	Low	Low
Zebin et al. (2019) [73]	High	Unclear	Unclear	High	Low	High	Unclear	High	High
Zhang & Kuo (2024) [74]	Low	Unclear	Low	Unclear	Low	Low	Low	Unclear	Low
Zhang et al. (2024) [75]	Low	Low	High	High	Low	Low	Low	High	Low
Zhao & Luo (2025) [76]	Unclear	Unclear	Unclear	High	Low	High	Unclear	Unclear	Unclear

Supplementary Table S5. Meta-analysis dataset (AUROC).

Author	Year	N	AUROC	Lower 95% CI	Upper 95% CI	SE	ICU Setting (Surgical / General)
Chen et al. [50]	2021	106	0.837	0.766	0.908	0.0362	Surgical
Grovu et al. [53]	2023	5 831	0.89	0.88	0.93	0.0128	General
Iwase et al. [58]	2022	2550	0.889	0.849	0.936	0.0222	General
Lefering & Waydhas [60]	2024	108178	0.903	0.9	0.905	0.0013	Surgical
Nallabasannagari et al. [64]	2020	42818	0.9178	0.9062	0.9285	0.0057	General
Shi et al. [68]	2024	464	0.87	0.831	0.908	0.0196	General
Wang et al. [71]	2022	109	0.8828	0.8572	0.9284	0.0182	Surgical
Weissman et al. [72]	2018	25947	0.89	0.88	0.9	0.0051	General
Zhang et al. [75]	2024	266	0.933	0.902	0.963	0.0156	Surgical
Zhao & Luo [76]	2025	227	0.928	0.895	0.961	0.0168	Surgical
